# Introduction to the Wayne Getz Festschrift

**DOI:** 10.1186/s40462-023-00441-x

**Published:** 2024-01-29

**Authors:** George Wittemyer, Sadie J. Ryan

**Affiliations:** 1 Department of Fish, Wildlife and Conservation Biology, Colorado State University, 80523 Fort Collins, CO USA; 2 Quantitative Disease Ecology and Conservation (QDEC) Lab, Department of Geography and the Emerging Pathogens Institute, University of Florida, 32611 Gainesville, FL USA; 3 School of Life Sciences, University of KwaZulu-Natal, Durban, South Africa

As the challenges to ecological systems from human induced landscape and climatic changes have begun to alter ecological systems globally, the need for ecological information about the responses to such changes has grown rapidly. Animal movement data has become a key resource to understand and quantify the changes ecological systems are experiencing, as well as understand baseline (possibly normative) ecological dynamics. Animal movement is one of the most sensitive metrics of animal behavior that can be collected relatively easily over extended time frames and across numerous individuals. The spatio-temporal dimension of movement data allows its application to myriad questions, from determining individual strategies to predicting population distributions. However, many aspects of the drivers of movement processes are opaque, nuanced or dynamic. As a result, creative research approaches are necessary to realize the full value of movement data, particularly in relation to its application to the complex ecological challenges we are facing today.

To meet this challenge, the discipline of movement ecology has grown exponentially over the past decades spurred by technical advances and seminal works in the field that catalyzed new ideas, thinking and approaches to studying and deciphering organismal movement. Prof. Wayne M. Getz, as a catalyst and innovator for this discipline, has been at the forefront of this growth, pushing ideas on how to conceptualize the discipline of movement ecology, the development and application of novel analytical approaches to glean information from movement data, and, most fundamentally, the questions and ecological concepts to which the applications of movement data are most relevant. Following these decades of contributions, Prof. Wayne M. Getz has recently announced his retirement from the editorial board of the journal Movement Ecology. His retirement represents the culmination of deep contributions to the field including in his foundational role in the development and establishment of Movement Ecology as a preeminent journal on organismal movement.

To recognize his excellent service to the journal and field, we have catalyzed a Special Feature in Movement Ecology, the Wayne Getz Festschrift. The aim of this special feature is to celebrate his storied career, given that few individuals have catalyzed as much thought, training and development in the discipline. Contributions to this feature were limited to collaborators, students, mentors and mentees of Prof. Getz. Given his broad contributions, the special feature was not topically limited beyond the requirement that all studies will be directly and closely linked to movement ecology and the contributions captured the essential focal areas of his work in the discipline.

While Wayne’s contributions to the field were many and varied, his work tended to address three core foci, (1) the development of novel applications to estimate space use and its structure, (2) the deconstruction of animal movement into its elemental parts to better understand the movement process, and (3) the characterization of animal interactions as captured through movement data. These foci are strongly represented in the contributions composing the Festschift (Table 1). Vissat et al. 2023 in *Categorizing the geometry of animal diel movement patterns with examples from high resolution barn owl tracking* [6] and Thie et al. 2023 *Linking migration and microbiota at a major stopover site in a long-distance avian migrant* [5] deconstruct the movement paths of their study organisms to characterize foraging behavior and stop-over ecology underpinning survival and physiological indices. These works highlight new insights on behavior from assessing fine scale movement patterns using ultra high resolution tracking data not commonly being studied in most systems. Dougherty et al. 2022 *A framework for integrating inferred movement behavior into disease risk models* [2] and Huang et al. 2023 *Variation in herbivore space use: comparing two savanna ecosystems with different anthrax outbreak patterns* [4] look at the utility of applying movement date to determine the spatial patterns of host-pathogen interactions on dynamics landscapes. These works demonstrate new insights gained through integrating spatial and disease ecology. Cain et al. 2023 *Movement predictability of individual barn owls facilitates estimation of home range size and survival* [1] relate movement patterns to space use and ultimately survival, in an in depth assessment of how movement reflects personality traits that structure fitness. In Gilbertson et al. 2023 *Agricultural land use shapes dispersal in white-tailed deer (Odocoileus virginianus)* [3], a comprehensive description of dispersal in white-tailed deer as a function of landscape features reveals the role of agriculture in fine scale movement. This work demonstrated the implications such movement strategies can have for managing Chronic Wasting Disease (CWD). Finally, Wall et al. 2023 *Land use drives differential resource selection by African Elephants in the Greater Mara Ecosystem, Kenya* [7] takes a novel approach to comparing elephant resource selection functions derived for different human land uses in a contiguous area.

**Table 1:** Special feature articles

Land use drives differential resource selection by African Elephants in the Greater Mara Ecosystem, Kenya
Variation in herbivore space use comparing two savanna ecosystems with different anthrax outbreak patterns in southern Africa
Categorizing the geometry of animal diel movement patterns with examples from high-resolution barn owl tracking
Movement predictability of individual barn owls facilitates estimation of home range size and survival
Linking migration and microbiota at a major stopover site in a long-distance avian migrant
Agricultural land use shapes dispersal in white-tailed deer (Odocoileus virginianus)
A framework for integrating inferred movement behavior into disease risk models

All papers represent examples of using movement behavior to glean unique insights into ecological processes underpinning ecological theory or key applied activities and, therefore, represent the key advances provided by Prof. Getz’s efforts in this area.

## Data Availability

No datasets were generated or analysed during the current study.
